# “Allopath” vs. Any Other “Path,”

**Published:** 1878-03-20

**Authors:** 


### "ALLOPATH" VS. ANY OTHER "PATH."

The term "Allopath" is much used as an opprobrious epithet in some quarters. On a certain occasion, recently, a gentleman of the eclectic persuasion publicly asserted that before his conversion, and while an "Allopath," he had killed his thousands (?). Thousands make regiments, and regiments an army. He was a mighty warrior before the Lord—a perfect angel of death. He does better under a better system. He now only slays his hundreds, we suppose. We congratulate him. Some men are doctors independent of systems, but a correct system helps wonderfully; and all good physicians are true eclectics. We know an "Allopath"—who is an "Allopath" still—who has had better luck in *selecting* his remedies than our eclectic friend. He does not slay his thousands under that system. He has considerable practice in a large city. He is a student of medical literature, and endeavors to keep himself up to the times. He rejects no remedy that any system advances. During two years and a half, he has lost three patients. He is an "Allopath," but does not abuse eclectics.

The *Arkansas Medical Record*, a new and neat little journal, is on our table; edited by Dr. J. I. Hale, of Little Rock. Success to you, brother Hale.

MEDICAL ASSOCIATION OF GEORGIA.—The Medical Association of Georgia will meet in Atlanta, on Wednesday, the 17th day of April, next.

We learn that a Committee of Arrangements has been appointed for the reception of the members, and that the society will assemble in the Senate chamber at 11 o'clock.

---

THE MEDICO-CHIRURGICAL ASSOCIATION OF ATLANTA, will hold its regular meetings hereafter on the 1st and 3d Monday evenings of each month.

---

THE *Michigan Medical News*, a snug little semi-monthly, has been sent us for exchange. It has quite an able corps of editors. J. J. Mulheron, M. D., managing editor.

---

PINKLYVILLE, Oregon Co., Mo.,
March 5, 1878.

*Dr. R. C. Word:*

Will you, or one of your co-editors, or some one of the RECORD'S correspondents, please give us an article on "so-called" typhoid-pneumonia—the capillary bronchitis of Flint's practice? I wish, particularly, for the views of a competent Southern practitioner as to pathology and treatment. The disease is very prevalent and very fatal in this section—terminating in death on the 5th—rarely on the 3d, 7th and 12th.

Very respectfully,
S. H. ANDERSON, M. D.

[Would be pleased to have a practical response by some of our readers to the enquiries of Dr. A. —ED.]

